# Demonstrating proof of concept for value-based agreements in Europe: two real-world cases

**DOI:** 10.1017/S0266462323000260

**Published:** 2023-05-22

**Authors:** Elizabeth A. Griffiths, Onivefu E. Odelade, Joana Gostkorzewicz, Luis Cordero

**Affiliations:** 1 AstraZeneca, Cambridge, UK; 2 AstraZeneca, Madrid, Spain; 3 Independent Consultant, Madrid, Spain

**Keywords:** Reimbursement mechanisms, access to medicines, value-based purchasing, proof of concept study, financial risk sharing

## Abstract

**Objectives:**

Value-based agreements (VBAs) link access, reimbursement, or price to the real-world usage and impact of a medicine, thereby enabling patient access while reducing clinical or financial uncertainty for the payer. VBAs have the potential to support improved patient outcomes, given the value-oriented approach to care, and lead to overall savings, while enabling payers to share risk and reduce uncertainty.

**Methods:**

This commentary outlines the key challenges, enablers, and a framework for successful implementation by comparing the experience of two VBAs for AstraZeneca medicines, aiming to increase confidence in their future use.

**Results:**

Engagement by payers, manufacturers, physicians, and provider institutions, and robust data collection systems that are accessible, simple to use, and add little burden to physicians were key to successfully negotiating a VBA that worked for all stakeholders. In both country systems, a legal/policy framework enabled innovative contracting.

**Conclusions:**

These examples demonstrate proof of concept for VBA implementation in different settings, and may inform future VBAs.

## Introduction

Scientific progress and pharmaceutical innovation continue to deliver new treatments that offer significant benefits to patients and healthcare systems. However, uncertainty around the magnitude and duration of the clinical benefit of novel treatments may pose challenges to current pricing and reimbursement models and the associated health technology assessment (HTA), in terms of budget impact affordability, and value for money. This is particularly true for indications where high patient unmet need warrants early access but collecting clinical evidence is challenging.

One solution to these access challenges is value-based agreements (VBAs). A VBA is an agreement between a manufacturer and a government/payer to reimburse a medicine under certain conditions. A VBA can be categorized broadly as a financial-based agreement (FBA), an outcome-based agreement (OBA), or a service-based agreement (SBA) (1;2). Under an OBA, the price of the medicine is determined by how well it performs. If a treatment works as intended, the patient and healthcare system benefit, and therefore the agreed price is paid. If a treatment falls short of what was expected, payers pay less or not at all. All VBAs, but particularly OBAs, support a move from a focus on volume to a focus on value. There is a large body of literature setting out different taxonomies and documenting the theoretical use of VBAs, but relatively little on how they are used in practice (3).

VBAs also have a potentially important impact on HTA. This can occur in several different ways. At a national level, VBAs can, in some circumstances, be vehicles for addressing uncertainties identified in the HTA process (e.g., when agreed after the initial assessment) and could subsequently be important in any reassessment. At a regional level, VBAs can be used to address uncertainties about whether national assessment applies in the regional context. At the local level, and under the appropriate circumstances, VBAs can be used to develop evidence that can subsequently feed into national assessments (e.g., by informing local coverage to generate and contribute data to a national assessment).

Despite the rising popularity of VBAs and the increasing literature on the number and types of VBAs, few publications provide actual examples of how these work and the benefits they deliver. The aim of this commentary is to address this gap, improve transparency on the workings of VBAs, and outline the key challenges and enablers, and a framework for successful implementation, based on a comparison of the experience with two VBAs for AstraZeneca medicines that have been agreed in two European countries (as described in Table 1).

**Table 1.** Detailed description of each VBA

	Case study 1. Dapagliflozin for diabetes patients requiring intensification in UK CCG	Case study 2. Gefitinib for patients with NSCLC in Catalonia, Spain
Type	Patients who meet the inclusion criteria (below) initiate treatment at list price, with a partial rebate given for their treatment cost, effectively reducing the price for 6 months After the first 6 months of treatment, clinical response is assessed against an agreed reduction in HbA1c or achievement of a HbA1c target. If the target is met, the patient continues at full price; if not met, patients can discontinue at no further cost, if clinically appropriate	Treatment is initially paid for in full by the health care provider for the qualifying patients, but for those patients who have not achieved the specified outcome at the time of the assessment, the pharmaceutical company provides a rebate to the health care provider covering the entire cost of the treatment administered up to the evaluation point
Duration	Two-year pilot period with potential for extension in 2023 as well as expansion to other CCGs	Two-year pilot period, with an evaluation period from June 2011 (inclusion of first patient) to October 2013, which was later extended
Eligibility criteria	Currently on Metformin alone No history of other antidiabetic agents Uncontrolled HbA1c (>7.5%) Renal function: GFR > 60 ml/min Baseline HbA1c within 3 months prior to scheme initiation Meets all medicine license label requirements	The diagnostic, eligibility, and response criteria were those determined by the ICO clinical practice guidelines and by ICO specialists: newly diagnosed or already diagnosed EGFR-mutation positive NSCLC patients Confirmed EGFR-mutation negative patients were not eligible
Outcome measure	HbA1c response (target informed by clinical guidelines and trial data)	According to RECIST criteria
Success evaluation criteria	Quarterly: Scheme uptake: n. patients on scheme, and % of eligible patients % of patients reviewed at 6 months % of responders (patients meeting the sixth month HbA1c response criteria) Discontinuation rates At 2 years: Patient outcomes – HbA1c response and additional endpoints (e.g., weight, blood pressure) Longer-term persistence on therapy After 2 years, scheme continuation based on: NHS and manufacturer willingness Satisfaction with scheme Environmental factors Administrative and resource burden	Patients who do not meet the criteria (i.e., eligible patient characteristics, measure of treatment response at established time points without missing variables or registry errors) are excluded from the scheme According to the treatment variables, patients are classified into one of four subgroups: *responsive* (i.e., treatment variables meet contractual definition of clinical response) *nonresponsive* (i.e., treatment variables do not meet contractual definition of clinical response) *awaiting results* (i.e., patients who have not yet reached the point for treatment follow-up) *others* (i.e., patients who discontinue the treatment because of reasons that are not covered by the agreement, for instance by patient’s choice)
Data tracking infrastructure and capability	Data is collected using a third-party data extraction company, which will install a software currently used for other data-extraction purposes in the NHS	The implementation of agreements in Catalonia has been largely supported by the use of patient registries and online databases to monitor drug use and cost The Patient and Hospital Outpatient Drugs Treatment Registry (RPT-MHDA) is a broad, specific, and centralized online registry for all SISCAT hospitals designed to systematically collect information on the use of innovative hospital outpatient drugs under conditions of routine clinical practice Data analysis is performed according to standards of observational studies and internal standard operating procedures
Data collected	Evidence generation is an intrinsic part of scheme, with a number of key outcomes assessed: Baseline characteristics, HbA1c, weight, blood pressure, cholesterol, and antidiabetic medications	The RPT-MHDA provides information on basic patient data (personal identification code, age, sex), treatment (drug identification, therapeutic indication, initiation and termination date, prescribing hospital), baseline patient characteristics, follow-up clinical variables, and discontinuation variables
Payment terms	The system generates a summary (invoice) of payments due to the CCG, based on prescribing of the medicine in the scheme-eligible cohort and achievement of target criteria based on HbA1c measures recorded within the patient records	The Invoicing for Health Services Application (FSS) is an online database that monitors hospital outpatient drugs expenditure by registering and collecting billing data from each hospital and patient on a monthly basis
Data ownership	All extracted data and audit results always remain under the control of the requesting practice Only aggregate-level data is available to the CCG and manufacturer without any patient-level outcomes	Only ICO/CatSalut had full access to individualized patient data. Hospital access is restricted to patient data for patients who have been treated in that hospital Manufacturers do not have access to patients’ data, in alignment with the European General Data Protection Regulation. Descriptive analyses on health and financial outcomes are shared with manufacturers prior to the invoicing process, but the results of these analyses are aggregated (by hospital and as a sum of all the participating hospitals) for anonymization purposes
Planned evaluation	The review of patients would have been at a 6-month period in line with clinical practice, at which point payments to the CCG would also have been made The evaluation of the scheme itself would most likely have started at the end of the first year to 18 months, led by the manufacturer to determine scheme success and inform continuation of the scheme The pilot would also likely have been evaluated by the CCG itself to determine whether financially and in terms of patient outcomes the scheme is successful and should be expanded	A commission formed by the three stakeholders periodically assessed the results Preliminary results were sent to the respective hospitals, which were required to validate or amend their patients’ data via the online registry and clarify any remaining doubts if needed Three time periods were compared: up to week 8, between week 8 and week 16 in patients who were stable at week 8, and up to the end of treatment in patients who had not withdrawn from treatment prior to week 16

*Source:* Interview with former CCG representative and interviews with former payers involved in the VBAs.

CCG, clinical commissioning group; EGFR, epidermal growth factor receptor; GFR, glomerular filtration rate; HbA1c, hemoglobin A1c; ICO, Catalan Institute of Oncology; NHS, National Health Service; NSCLC, nonsmall cell lung cancer; RECIST, response evaluation criteria in solid tumors; SISCAT, Integrated Public Healthcare System of Catalonia; VBA, value-based agreement.

### VBAs in the United Kingdom and Spain

Two examples of VBAs, specifically OBAs, in the United Kingdom (UK) and Spain, were selected to provide lessons across different healthcare systems. These also cover two different therapeutic areas with different challenges. In both cases, the objective of the VBA was to address uncertainty regarding the real-world performance of the treatment and value for money (in terms of cost-effectiveness and budget impact). For example, in the Spanish case, there was uncertainty regarding the level of waste due to inappropriate decision making (i.e., the prescribing of less effective treatments) in real clinical practice, and how to ensure that a new medicine would be used in the most appropriate patients, as documented in multiple HTA reports (4).

The VBA in the UK, contracted with a clinical commissioning group (CCG), was for dapagliflozin, a sodium-glucose cotransporter 2 inhibitor (SGLT2i) for the treatment of type 2 diabetes mellitus (T2DM). The CCG signed the agreement in 2020 and planned rollout to all general practitioner (GP) practices in the CCG following a short test phase in two pilot practices. The agreement was for 2 years with an option for an extension. Under the scheme, patients would initiate treatment at a reduced price, realized via a partial rebate for their treatment cost. If after 6 months, patients achieved an agreed reduction in hemoglobin A1c (HbA1c) or an HbA1c target, they would continue treatment at the list price, reflecting the value delivered. This is an example of a VBA based on the performance of surrogate endpoints (rather than directly measuring how a patient feels, functions, or survives). Surrogate endpoints may be used if the timely measurement of differences in morbidity or mortality is challenging or unfeasible, and there is solid evidence for the close correlation between the surrogate endpoint and the desired outcome (5).

Implementation of the UK VBA was delayed due to the COVID-19 pandemic and CCG restructuring, which also impacted the rollout of other quality improvement initiatives in the National Health Service (NHS). National Institute for Health and Care Excellence (NICE) guidelines on managing T2DM in adults were also updated during this time, recommending SGLT2i use earlier in the treatment pathway and in a wider cohort of patients. These changes were aligned with the objectives of the scheme. However, a mutual decision was reached to terminate the contract and reconsider how the scheme could be modified to provide greater value for money in the restructured NHS organization and support implementation of the updated NICE guidelines on T2DM. Hence, although successfully negotiated, the VBA was not applied in practice. An alternative approach that addresses the same issues that motivated the scheme is still under discussion.

The VBA in Catalonia, Spain, was a pilot scheme for gefitinib, an epidermal growth factor receptor (EGFR) inhibitor, for use in newly diagnosed or already diagnosed EGFR mutation-positive nonsmall-cell lung cancer (NSCLC) patients, and was managed by the Catalan Institute of Oncology (ICO) and the Catalan Health Service (CatSalut) (6). It was the first such scheme in Catalonia (7). Outcomes according to response evaluation criteria in solid tumors (RECIST), a standard framework for evaluating tumor response to treatment (e.g., smaller, same, larger), were determined up to week 8, between week 8 and week 16 in patients who were stable at week 8, and up to the end of treatment in patients who had not withdrawn from treatment prior to week 16. Treatment was initially paid for by the healthcare provider. AstraZeneca reimbursed the cost of the treatment for nonresponders. The pilot scheme ran from June 2011 to October 2013.

To reflect both the manufacturer’s and payers’ perspectives, we conducted six semi-structured interviews (completed between June and October 2021) with those involved in the development of the VBA: the manufacturer and the payers in the UK and Spain. The interviews were conducted to understand the goals and objectives of each VBA, its structure, challenges with its negotiation or implementation, key enablers for implementation, and planned evaluation or observed benefits, discussed in the following section.

## Results

### Context and Objectives

The VBA in the UK was negotiated in the context of the Quality, Innovation, Productivity, and Prevention (QIPP) program, with ambitious CCG savings targets, aiming to address a funding gap of £30 billion (€35.7 billion as estimated by the Nuffield Trust and NHS England) by 2021 (8). Given these budgetary constraints, wider use of the newer SGLT2i was seen as challenging. The CCG and the manufacturer established a VBA with the shared objective of removing financial considerations from the prescribing decision, allowing the medicine to be prescribed earlier in the diabetes treatment pathway (preempting the new guidelines) where clinically appropriate, with the expectation of improved patient outcomes and value for money.

The VBA in Spain was the first in Catalonia. It was implemented in the context of Catalonia’s 2011 Health Plan and affordability challenges with access to innovative medicines. The plan aimed to implement results-oriented payment systems, sharing risk with pharmaceutical companies, and to structure the system around patient needs, efficiency, and equitable resource allocation (9). Specifically, the VBA for gefitinib was proposed by ICO and the Catalonian Pharmacy Commission, with the objective of addressing uncertainties about its effectiveness, cost-effectiveness, and budget impact in the Catalonian population. Gefitinib has an associated biomarker for EGFR mutations, enabling the definition of a pragmatic “payment-by-result” scheme.

An important objective of both schemes was to streamline the process for and increase the experience of payers and manufacturers with VBAs to improve patient access, while assessing real-world effectiveness.

### Administrative and Legal Framework

Both VBAs underwent a robust review process and had strict legal and compliance safeguards. The schemes were reviewed by different stakeholders within the local authorities responsible for their contracting (the CCG’s Medicines Optimization workstream and the Pharmaco-therapeutic Committee and ICO/CatSalut in Catalonia), and their approval and buy-in were ensured. The company’s legal department ensured compliance with competition law and patient confidentiality safeguards. Patient eligibility criteria were established based on input from clinicians; it was agreed that these should be in line with the label indication and based on established clinical practice, to ensure that the use of the medicine was not favored over alternatives. In both cases, the agreement was based on detailed discussions, although the origins of the design differed between the examples. In the UK, the pharmaceutical company proposed the overall structure of the scheme, including the response criteria and points of evaluation for dapagliflozin. In the case of gefitinib, ICO oncologists proposed the scheme, eligibility, and response criteria.

### Data Collection

The data collection process varied, reflecting differences in the healthcare settings, the therapy areas, and the available data infrastructures at the time of implementation. However, in both cases, an important objective was to minimize the additional administrative burden on healthcare professionals (HCPs) by integrating data collection into existing systems. The dapagliflozin VBA relied on automatic patient-level data collection through software already installed and in use in most of the GP practices, so the administrative burden on GPs was expected to be minimal. The data available to AstraZeneca and the CCG for analysis would have been aggregated and would not allow for identification of individual patients. For gefitinib, the data was collected in an existing electronic prescribing system in all ICO hospitals. Electronic data collection had already been a requirement for prescribing innovative oncology drugs since 2006 (10), so the scheme added little additional burden. Furthermore, the only additional costs resulted from the need to follow-up on the agreement (two meetings in 2 years), and the administrative costs of the reimbursements (6). Data ownership was also an area for discussion. In the UK, data ownership agreements were also drafted and agreed by the CCG Privacy Officer. Ownership remained with the GP practices for the dapagliflozin VBA and with hospitals for the gefitinib VBA.

### Evaluation and Next Steps

From the outset, evaluations of the VBAs informed the design. The data collection supported different decision points. As set out in Table 2, this differs for the two examples. The reimbursed amount (or rebate) under the dapagliflozin scheme was to be determined automatically every 6 months, based on the analysis of anonymized and aggregated data collected through the digital platform led by the CCG. An evaluation of the scheme itself was scheduled after 12–18 months, to be led by the manufacturer, in order to determine its success and inform decisions regarding whether to continue the VBA. Meanwhile, the rebate for gefitinib was determined by a follow-up committee (comprising the three stakeholders involved, including both ICO and AstraZeneca) at three time periods, based on patient-blinded data collected in the electronic system.

**Table 2.** Framework for the successful implementation of VBAs

Theme	VBA enabler	Details
Willingness	1. Buy-in	Appreciation of need for innovative access solutions Willingness of all stakeholders to collaborate to overcome implementation barriers, including buy-in from physicians (achieved, e.g., by including physicians in the VBA process)
Ability	2. Value assessment	Mechanism (e.g., HTA) enabling fair assessment of medicine value at the indication level A mutually agreed process for the evaluation of the VBA and for the subsequent dissemination of best practices
	3. Trackable usage/outcomes	Robust data collection protocol and procedures Trusted data to inform contract development, agreement, and implementation Identifiable and pragmatically trackable outcomes data where required, while maintaining patient confidentiality Feasible tracking system acceptable to the payer, physician, and health system
	4. Contract/billing infrastructure	Infrastructure and processes enabling calculation and processing of payments or rebates
	5. Legal framework and policy landscape	Legal/regulatory policies permitting innovative contracting (e.g., net price confidentiality) Policy supporting appropriate data capture and use to support contracting

HTA, health technology assessment; VBA, value-based agreement.

The influence of VBAs on the HTA process is nuanced and depends on the type of VBA and where it is to be implemented. The local VBAs described here were not a direct result of a national HTA process but were intended (by manufacturers and payers) to address uncertainties at the regional level or to develop data from pilots that could subsequently be used to update national guidelines, payment policies, and/or HTAs.

In both cases, the schemes were novel and were seen as pilots by the stakeholders involved. It was agreed in advance that geographical expansion of the UK VBA would have depended on whether the scheme met its objectives, as well as on the financial and administrative burden on both parties. The representative from the CCG highlighted the importance of best practice sharing with other CCGs across the UK to facilitate its wider implementation. The termination of the UK VBA illustrates the need to consider broader environmental changes (such as guidelines, regulations, policies, health system reforms, new evidence, and availability of other medicines) when determining the next steps.

In Spain, the success of the scheme was evaluated by the follow-up committee. It was deemed to be successful, as the effectiveness of gefitinib was shown to be similar to that demonstrated in the clinical trials; overall, thirty of forty-one patients in the scheme (73 percent) were assessed as having an adequate response at week 16, compared with a response rate of >70 percent in the clinical trials (6). The scheme increased confidence in the generalizability of the clinical trial data to clinical practice, and helped to facilitate access to an innovative medicine. The VBA also had direct benefits in terms of treatment cost savings compared with the traditional purchasing scenario (with savings of around €880 per patient and €36,000 in total, which translated to a 4.15 percent reduction in billing) and indirect benefits by improving clinical practice processes (facilitating faster access to bronchoscopies and scanning). This experience informed the development of standardized guidelines in Catalonia for VBAs implementation of VBAs (11), and fifteen VBAs subsequently were implemented between 2016 and 2019 (7).

### Expected or Observed Benefits, Challenges, and an Implementation Framework

Concerns have been expressed that VBAs might impose undue administrative burdens, or benefit one stakeholder over another (12). Therefore, it is important to determine whether the manufacturer and the payer agreed on the observed benefits of these two schemes. In both examples, drawing on the interviews, we find there was considerable agreement between the manufacturer representatives and payers involved in the development of the VBAs, regarding the following benefits:

- VBAs can help to foster efficient use of limited healthcare resources while giving patients access to innovative medicines, thereby improving patient outcomes and quality of care. Both payers believed that the additional cost of implementing the VBA was outweighed by the savings generated and by improved patient outcomes in clinical practice (or would have been, in the case of the UK scheme being implemented).
- The data collected can inform treatment guidelines, ensuring more appropriate use, in line with medicines optimization principles. A potential benefit of local or regional agreements, where data can be gathered in pilot programs, is that subsequently, it can feed into national HTA processes. This was the intention of the UK OBA. In addition, manufacturers can benefit from the use of real-world data in supplementing the clinical development data package, updating and improving regulatory license applications, and informing commercial strategies.
- VBAs might enable payers to better manage healthcare budgets associated with hospitals or practices. The requirement for outcomes monitoring through data collection may provide more timely information that allows nonresponding patients to switch to more effective treatment alternatives sooner, thereby reducing the use of ineffective treatments and avoiding unnecessary treatment cost.
- VBAs enable payers and companies to share financial risk related to uncertainties about the effectiveness of a medicine and its utilization, while also generating local real-world data and experience with the new intervention in the local context. The possibility of a medicine performing much better or worse than expected should be considered when agreeing the VBA terms.
- VBAs support patients and HCPs in making informed choices, reducing ineffective health interventions and avoidable complications, and providing evidence for continuous advancement on treatment pathways.
- Implementing a pilot can increase experience with VBAs, and enable payers to set up the necessary decision-making processes and data collection infrastructure to facilitate the smoother implementation of similar schemes in future. Payers and manufacturers reported that it can also help build trust.

However, VBAs can present challenges. Payers and manufacturer representatives interviewed for this commentary identified the following challenges:

- VBAs should be used where data infrastructure (e.g., electronic patient records) enables the collection and subsequent analysis of patient outcomes without additional administrative burden for payers and physicians. Where the data infrastructure is not in place, it will need to be set up; this is a time-consuming and resource-intensive process requiring collaboration between stakeholders, which includes establishing ownership of the data and a process to maintain confidentiality.
- Trust and willingness can take time to establish. In the UK and Spain, a key barrier to agreeing on and implementing VBAs was the limited experience of both parties in working together on VBAs. It took time to develop a dialogue between the relevant stakeholders and to determine partners willing to collaborate on scheme design. Policy initiatives (such as the Catalonia Regional Health Plan) can provide a useful platform for dialogue.
- The use of VBAs needs to be designed considering the wider set of incentives affecting different stakeholders. Depending on the healthcare system, these may include incentives for providers to use value-based contracting, or may account for prescribing incentives at the hospital or practice level that might conflict with the VBA objectives (as discussed in the UK example).

Further, from the manufacturer’s perspective, these VBAs provide proof of concept for VBAs at the local level, and informed future scalability. The stakeholders interviewed highlighted the importance of providing timely access to novel medicines, thereby improving patient outcomes and helping to sustain future investment in innovation. In the specific cases discussed in this article, the OBAs allowed (or would have allowed) new treatments to be introduced more quickly under appropriate and agreed conditions of use; under other circumstances, access may have been delayed or restricted.

Based on these challenges and benefits, the payers and manufacturers highlighted a set of key enablers for the successful implementation of VBAs, which are summarized in a framework in Table 2. This can serve as a guide to stakeholders in negotiating and implementing VBAs (with supporting quotations in Supplementary Table 1).

## Conclusions

There is increasing consensus that spending on healthcare interventions should be considered in the context of the value and outcomes delivered, and the impact on the wider healthcare system and society. Tracking treatment usage and outcomes over time allows us to better understand how to improve clinical decision making. It also gives payers greater certainty that they are paying for the real value of treatments to patients. Although challenging to implement across complex healthcare systems, VBAs can help to address these uncertainties by sharing the risks related to health outcomes and costs, while providing timely managed patient access.

We outlined the experience with two VBAs for two disease areas (diabetes and NSCLC) based on interviews with payers and manufacturers in the UK and Spain. All stakeholders highlighted the positive impact of VBAs to date in terms of potential improvements in clinical practice and patient outcomes given the value-oriented approach to care and potential savings. The NSCLC VBA helped share risk associated with uncertainty about gefitinib’s effectiveness, while the diabetes VBA was intended to facilitate dapagliflozin’s use earlier in the treatment pathway. Despite the differences in the disease areas and healthcare settings, the learnings from their experience converge on a similar set of challenges and enablers for the implementation of VBAs. There are some lessons for where and how to apply VBAs in the future. Engagement of core stakeholders (payers, manufacturers, and physicians) was critical; robust data collection systems that were accessible, were simple to use, and added little burden to physicians were also key to successfully negotiating a VBA that worked for all stakeholders. In the UK and Spanish healthcare systems, a legal/policy framework enabled innovative contracting, and existing incentives were considered and mitigated. If these preconditions are in place, different types of VBAs can help manufacturers and payers to enable patient access to promising interventions while sharing risk and reducing uncertainty by generating real-world evidence on effectiveness. This also provides the basis for assessing value, which may inform subsequent payment policies or HTAs regarding new and emerging therapies. Although these were regional examples, the experience can be extrapolated to other regions and to a national context. Finally, even though these VBAs were agreed sometime in the past, the lessons appear as relevant today.

A growing number of VBAs are being implemented in Europe (12). However, in practice, confidentiality around their elements, including the specifics of payment amounts and conditions, limits sharing best practices more widely. Given the completion of the pilot in Spain and the willingness of payers and manufacturers in Spain and the UK to share their experiences, these examples can be shared here to inform parties interested in VBAs in the future. We hope that this commentary broadens the practical knowledge base about planning and implementing VBAs. Increasing the transparency of some elements of VBAs may facilitate best practice sharing among payers and across healthcare systems.

## Supplementary material

For supplementary material accompanying this paper visit https://doi.org/10.1017/S0266462323000260. click here to view supplementary material
