# Supplementary material for: Demonstrating proof of concept for value-based agreements in Europe: two real-world cases
Source: Int J Technol Assess Health Care. 2023 May 22;39(1):e30. doi: 10.1017/S0266462323000260 (PMC11574532; doi:10.1017/S0266462323000260)
Supplement: Supplementary file 1 [file S0266462323000260sup001.docx]

**Supplementary Table 1: Quotes from the UK and Spanish payer interviews**

| Payer | Quotes |
| --- | --- |
| UK CCG Payer | “The objective specifically was to streamline processes so we could perhaps do it again in the future so it was not too labor-intensive for the CCG.”  “The treatment must continue to adhere to the diabetes treatment pathway which they already had in place. Another principle is that they must road test the scheme in the two pilot sites before rolling out more widely”  “We knew that we would have to approach GPs at each practice to ensure their buy-in. ... It would be a problem getting the scheme up and running if practices did not have full information about it beforehand, including time and opportunity to ask questions.”  “The scheme must not impact on GP time, there must be automatic data extraction with no IT problems.”  “Having the infrastructure in place was quite instrumental.”  “All the data is anonymized so it is safe, and the DPO [Data Protection Officer] went into it in quite a lot of detail and came away satisfied that there is no leakage of any protected data because of it being anonymized.” |
| Spanish Catalonia Payer | “ICO had multiple discussions with AstraZeneca – very good communication and good coordination with the Catalonian system, giving the same message to AstraZeneca, explaining why ICO wanted to do the scheme. AstraZeneca had a very positive attitude and understanding why ICO wanted to implement it.”  “Payers were looking for new ways but also wanted to learn and be sustainable and introduce new drugs in a period of economic crisis.”  “The oncologists were closely involved (together with managers and pharmacists at the hospital) in the conception of the scheme and were actively thinking of ways to deliver innovative oncology drugs.”  “The pilot gave them an opportunity to implement, evaluate, and provide evidence that such schemes are possible and successful.”  “The scheme achieved the objectives set out in the beginning. It gave them a tool to access innovative drugs and consequently enabled faster access for patients.”  “The scheme had collateral benefits related to the management of the process: [It] improved the time at which the scan needed to be done / the timing of the measuring of the outcomes. This is a challenge in cancer care... The scheme improved the process of bronchoscopies across hospitals based on data in all centers doing bronchoscopies and biomarker measuring.” |

*Abbreviations: CCG: Clinical Commissioning Group, GP: General Practitioner, ICO: Catalan Institute of Oncology, IT: Information Technology.*
